# Comparative study of the paraspinal muscles after OVF between the insufficient union and sufficient union using MRI

**DOI:** 10.1186/s12891-018-2064-0

**Published:** 2018-05-14

**Authors:** Marina Katsu, Tetsuro Ohba, Shigeto Ebata, Hirotaka Haro

**Affiliations:** Department of Orthopaedic Surgery, University of Yamanashi, 1110, Shimokato, Chuo, Yamanashi, 409-3898 Japan

**Keywords:** Osteoporotic vertebral fractures, Magnetic resonance imaging, Cross sectional area, Insufficient bone union, Fat infiltration rate, Paraspinal muscles

## Abstract

**Background:**

Identification of poor prognostic factors for OVF is important but has not yet been clearly established. Despite paraspinal muscles could play an important role in the etiology of OVF, what influence time-dependent changes in paraspinal muscles have after OVF, and the impact on conservative treatments for patients who have an OVF remain largely unknown. The purposes of this study were to (1) evaluate time-dependent changes of the paraspinal musculature using MRI after injury in patients with osteoporotic vertebral fractures (OVFs), and (2) compare paraspinal muscles between conservatively treated patients with OVF who have successful union and those failed to conservative treatment.

**Methods:**

A total of 115 consecutive patients who had sustained a recent OVF injury in the thoracolumbar region were assessed for eligibility using medical records and all required data were available from 90 patients who had been followed up for at least 6 months. Patients who needed to undergo surgery and patients who were diagnosed as having insufficient union after 6 months of follow-up were assigned to a group with insufficient union. Lumbar trunk parameters, relative cross-sectional area (rCSA) and proportion of fat infiltration (FI%) were calculated from MRI. To evaluate the time-dependent changes in the paraspinal muscle in patients after OVF injury, correlations between the timing of MRI and rCSA, FI% were determined. To clarify the impact of paraspinal muscles on the outcome of conservative treatments of patients with OVF, we compared rCSA between the groups.

**Results:**

Sixty-five patients were assigned to a group with insufficient union and 25 patients were assigned to a group with successful union. FI% of the multifidus and erector spinae in the group with insufficient union were significantly greater than in the group with union. The timing of MRI in relation to initial injury was significantly correlated with FI% of the multifidus and erector spinae. rCSA of the erector spinae was significantly larger in the group with successful union than in the group with insufficient union.

**Conclusions:**

These findings indicated a time-dependent increase of fatty degeneration of the multifidus and erector muscles, but no change in the rCSA and larger rCSAs of spinal erectors may play a role in successful union in patients with OVF.

**Electronic supplementary material:**

The online version of this article (10.1186/s12891-018-2064-0) contains supplementary material, which is available to authorized users.

## Background

Osteoporotic vertebral fractures (OVFs) increase overall mortality [1, 2], and are becoming increasingly common as the proportion of the population that is older continues to increase [3]. Described as stable spinal injuries, OVF are treated with conservative methods such as rest, immobilization, drug treatment and brace therapy [4]. However, these vertebral fractures sometimes fail to union, resulting in progressive collapse or pseudarthrosis and these patients often suffered from persistent back pain or neurological deficits [5]. The prevalence of insufficient union in elderly patients with OVFs ranges from 10 to 13.5% after conventional conservative treatments, resulting in marked reductions in the quality of life and activities of daily living of these patients [6–8]. Therefore, identification of risk factors associated with a poor prognosis for OVF is important but has not yet been clearly established.

Magnetic resonance imaging (MRI) plays crucial role in evaluation of spinal pathologies [9]. Recent studies have indicated the clinical importance of measuring the cross-sectional area (CSA) and proportion of fat infiltration (FI%) of paraspinal muscles using MRI to evaluate OVF or lumbar degenerative disease [10–15]. The CSA and FI% of paraspinal muscles correlate with spinal stability and alignment [11]. Additionally, trunk muscles could play an important role in the etiology of OVF based on how they affect spinal stability and vertebral strength via muscle–bone interactions [16]. However, what influence time-dependent changes in paraspinal muscles have after OVF, and the outcomes of conservative treatments for patients who have an OVF remain largely unknown.

The purpose of the present study was to (1) evaluate time-dependent changes of the paraspinal musculature after injury in patients with OVFs using MRI, and (2) compare paraspinal muscles between conservatively treated patients with OVF with successful union and insufficient union after conservative treatment.

## Methods

### Patient population

**Fig. 1:**
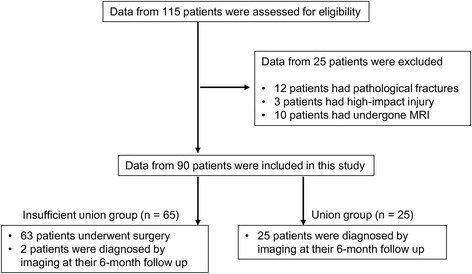
Flow diagram showing the enrollment and assignment of patients

A total of 115 consecutive patients older than 65 years who had sustained a recent OVF injury in the thoracolumbar region were assessed for eligibility using their medical records between 2010 and 2016 in this retrospective single-center cohort study. During their initial visit to our institute, a new vertebral fracture was diagnosed if the following were present: onset of back pain within 3 weeks before presentation, a deformed vertebral body visible on radiographs, and an abnormal intensity within the vertebral bodies visible with MRI had on the initial visit. Patients with multiple fracture, pathological fractures associated with a tumor, high-energy injury, or those with neurological deficits, or who had not undergone MRI of thoracolumbar spine were not eligible to participate in the present study. Initially, patients underwent conservative treatment determined by three board-certified spinal surgeons at a single institution and were followed-up for a minimum of 6 months. Treatment options, including the use of a brace or drug therapy, or both, were selected by the individual physicians based on their experience. All of the required data were available from 90 patients who had been followed up for at least 6 months (Fig. 1).

Insufficient union was diagnosed when patients underwent surgery within 6 months or were diagnosed based on intravertebral vacuum clefts on plain radiography or CT, or had apparent segmental motion on plain X-ray dynamic images (≥5° between supine and weight-bearing positions) after 6 months of follow-up, as assessed by two spine surgeons independently [17]. Surgical treatment was indicated for patients with progressive neurological deficits or continuous severe lower back pain (visual analog scale > 80/100 points) caused by insufficient vertebral bone union, or both. Sixty-three patients required surgical treatment because of insufficient bone union of their OVF for the present study.

#### Patient groups

Patients who needed to undergo surgery and patients who were diagnosed as having insufficient union after 6 months of follow-up were assigned to a group with insufficient union (Fig. 1). Average time from original injury to surgery was 4.2 ± 1.5 months. After 6 months of follow-up, based on radiographs of satisfactory bony union without associated severe pain, we assigned 25 patients to a group with successful union (Fig. 1).

### MRI protocol

The patients were placed in a supine position with the spine in a neutral position and a pillow was placed under their knees. Imaging was performed using a 3.0 T dual gradient superconducting MRI system (Discovery 750; GE Medical Systems, Milwaukee, WI, USA) with an 8-channel NeuroVascular-full neck coil, at a gradient strength of 40 mT/m, and a slew rate of 150 mT/m/ms. Sagittal and axial T1-weighted and T2-weighted MRI was performed routinely.

#### Measurement of the CSA and FI% of paraspinal muscles as visualized by MRI

**Fig. 2:**
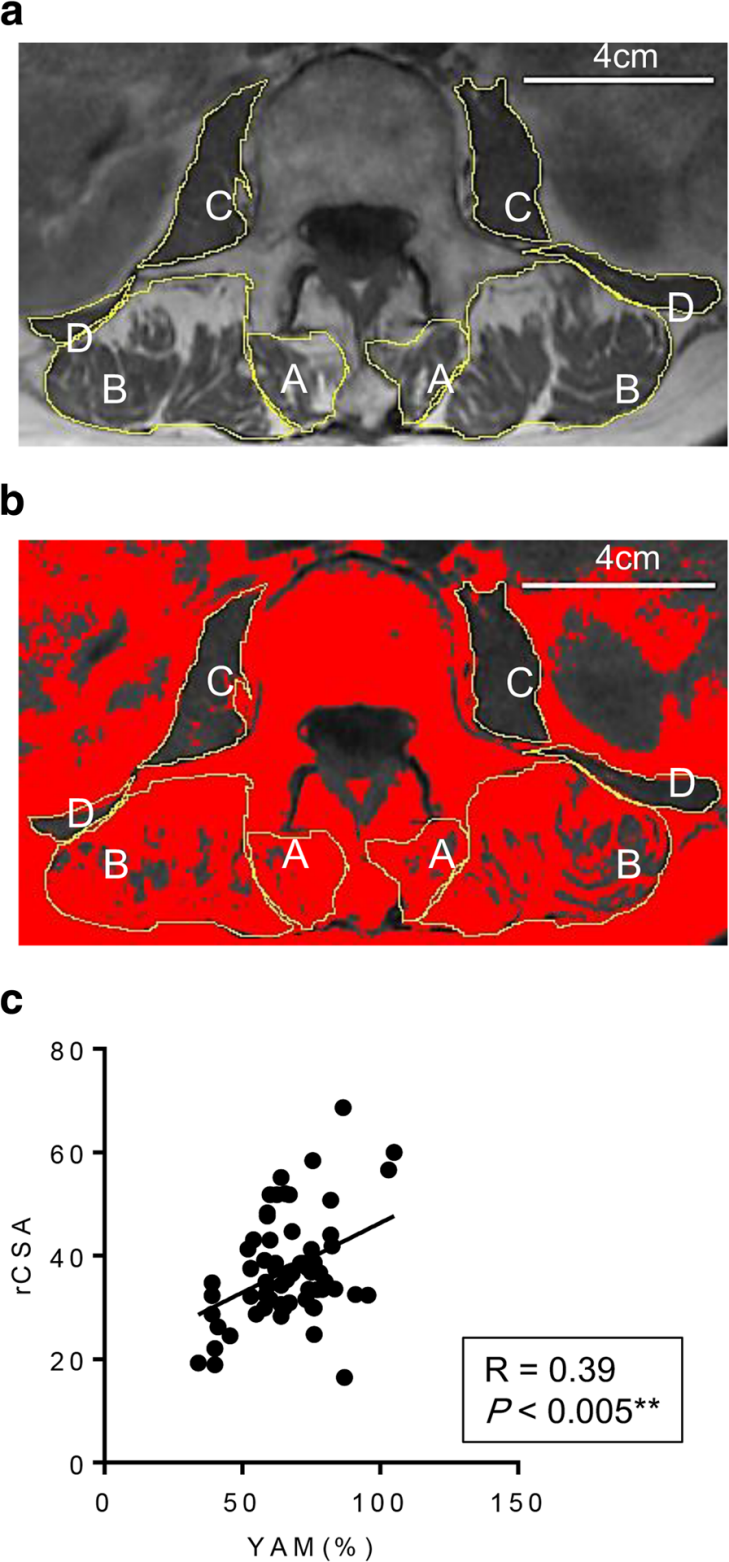
Measurement of the cross-sectional area on T1 axial image (**a**) and fatty infiltration of paraspinal muscles using Image J (**b**). A = multifidi; B = erector spinae C = psoas; D = quadratus lumborum. **c** Correlation between rCSA of total paraspinal muscles and BMD (YAM%)

All MRI measurements were made by an experienced orthopedic surgeon blinded to the clinical information and study hypothesis. Following a previously used method [18], the regions of interest (ROI) were defined by manual tracing of the fascial boundary of the following muscles on both sides of the spinal column to the superior endplate of the L3 vertebral body on T1 axial images: psoas major, multifidus (including rotatores lumborum spinae), spinal erectors (encompassing both the longissimus and iliocostalis), and quadratus lumborum muscles (Fig. 2A). The ROIs were analyzed, and histograms showing the signal intensity were generated using digitalized image processing software (Image J, National Institutes of Health, Bethesda, MD, USA). The FI% in the total CSA of both muscles was evaluated using a threshold technique. Briefly, the number of pixels representing intramuscular fatty tissue were distinguished using a threshold grayscale value of 120 [15]. Relative CSA (rCSA) was defined as the CSA of each of the muscles divided by the CSA of the L3 vertebral body [12, 18]. The investigator evaluated and re-evaluated MRI, 2 weeks apart, blinded to MRI identifiers, to assess the intraobserver reliability of the rCSA and FI% of paraspinal muscle measurement. The intraobserver error of FI% of all patients is shown in a Additional file 1.

### Data collection

For each patient who was treated for OVF, we searched the clinical records and laboratory database to record their basic characteristics, such as age, sex, body mass index (BMI), vertebral levels of injury, and which type of brace was used initially. Standardized bone mass density (BMD) measurements at the femoral neck were conducted using DXA (Lunar Prodigy; GE Medical Systems) within 4 weeks from the first visit. OVF were classified according to Genant’s semiquantitative criteria using plain X-ray images at the initial visit [19]. The severity of lumbar spinal stenosis seen on MRI was graded qualitatively according to the classification reported previously [20].

### Statistical analysis

All data are reported as mean ± SD. Data were analyzed using an unpaired *t* test and a Fisher exact test to determine significant differences. Pearson correlation coefficients between rCSA, FI%, BMD (YAM%), and timing of MRI after the initial injury were determined. All statistical calculations were conducted using Prism, version 6.0 (Graph Pad Software, La Jolla, CA). For all tests, *P* < 0.05 was considered significant (*P < 0.05, **P < 0.005, ***P < 0.0005, ****P < 0.0001).

## Results

We included 90 patients (58 women and 22 men) with an average age of 75.4 (65–88) years in the present study. Table 1 summarizes the characteristics of the patients, type of brace that was used initially and drug treatment for osteoporosis at their initial visit to our institute. There was no significant difference in the mean age, sex, BMD, and grading of LSS severity between the groups. The patients in the group with insufficient bony union had a more severe Genant grade of OVF than patients in the group with successful union. Overall, there were no significant differences in conservative management with hard braces or elastic braces between the groups. The rCSAs of total paraspinal muscles were positively correlated with BMD (YAM%) of all patients (Fig. 2c).

**Table 1:** Summary of the characteristics of each group

Characteristic	Insufficient union (*n* = 65)	Union (*n* = 25)	*p*
Age^a^ y, mean ± SD	75.9 ± 7.9	74.1 ± 11.2	0.41
BMI, kg/m^2^, mean ± SD	22.3 ± 2.9	22.4 ± 3.1	0.87
Sex, female/male	42/23	16/9	0.27
BMD (%YAM), mean ± SD	66.9 ± 16.3	69.2 ± 10.4	0.58
Level of injury			
Thoracolumbar junction level (T11–L2)	60 (92%)	22 (88%)	
Other level	5 (8%)	3 (12%)	
Genant’s classification			
Minimal fracture	9 (14%)	15 (60%)	< 0.0001****
Moderate fracture	15 (23%)	5 (20%)	1.00
Severe fracture	41 (63%)	5 (20%)	< 0.005***
Grading of LSS severity			
A	33 (50%)	11 (44%)	0.64
B	12 (19%)	5 (20%)	1.00
C	20 (31%)	8 (32%)	0.44
D	0	1 (4%)	0.27
Type of braces			
Custom-made hard braces	17 (26%)	6 (24%)	1.00
Elastic braces	31 (48%)	15 (60%)	0.35
No brace	17 (26%)	4 (16%)	0.41
Drug treatment			
Teriparatide	8 (12%)	4 (16%)	0.73
Bisphosphonate	11 (17%)	5 (20%)	0.76
Denosumab	5 (8%)	2 (8%)	1.00
SERMs	8 (12%)	3 (12%)	1.00
No treatment	30 (46%)	11 (44%)	1.00

*BMI* Body Mass Index, *BMD* bone mass density, *YAM* Young Adult Mean, *LSS* lumbar spinal stenosis, *n* number in group, *SERMs* selective estrogen receptor modulators

*P < 0.05, **P < 0.005, ***P < 0.0005, ****P < 0.0001

^a^Mean ± standard deviation (SD)

**Fig. 3:**
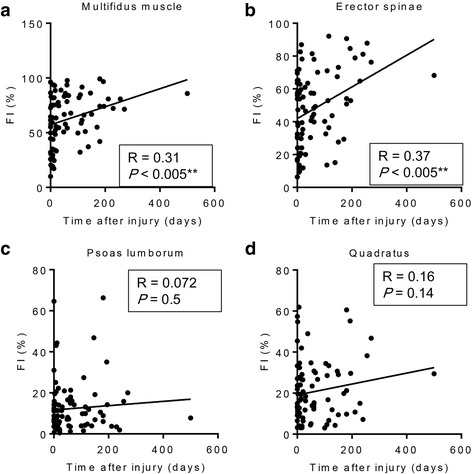
**a** Correlation between the timing of MRI after the initial OVF injury and the FI% of the (**a**) multifidus muscle, (**b**) erector spinae, (**c**) psoas lumborum, and (**d**) quadratus

**Fig. 4:**
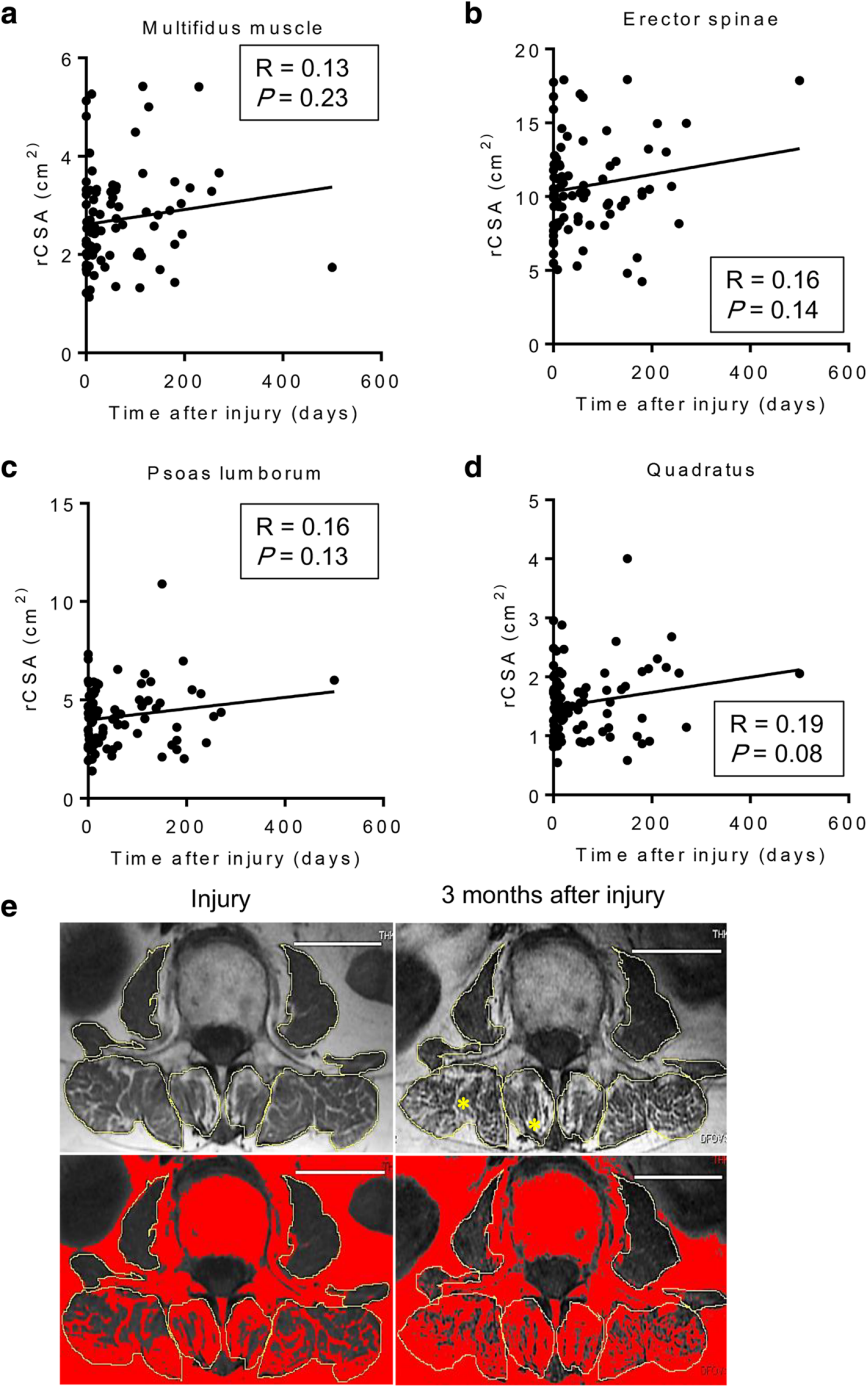
**a** Correlation between the timing of MRI after the initial OVF injury and the rCSA of the (**a**) multifidus muscle, (**b**) erector spinae, (**c**) psoas lumborum, and (**d**) quadratus. **e** Representative images of the time-dependent increase in FI%, but no change in rCSA of the multifidus and erector muscles

Despite that only patients who had undergone MRI were enrolled in this study, the timing of MRI after injury was indefinite and was assessed by three board certified spinal surgeons independently. The average time to obtain MRI in all cases was 64.1 ± 13.71 days after the initial injury. To evaluate the time-dependent changes in the paraspinal muscle in patients after OVF injury, correlations between the timing of MRI and rCSA, FI% were determined (Figs. 3 and 4). Timing of MRI after the initial injury was correlated with the FI% seen in the multifidus and erector spinae (Fig. 3a and b). By contrast, there was no correlation between the timing of MRI after the initial injury and the FI% of the psoas and quadratus, or rCSA of any paraspinal muscles (Fig. 3c and d, Fig. 4a–d). These findings indicated a time-dependent increase in FI% of the multifidus and erector muscles after injury, but no change in the rCSA of any paraspinal muscles (the paraspinal muscles of representative cases are shown in Fig. 4e).

**Fig. 5:**
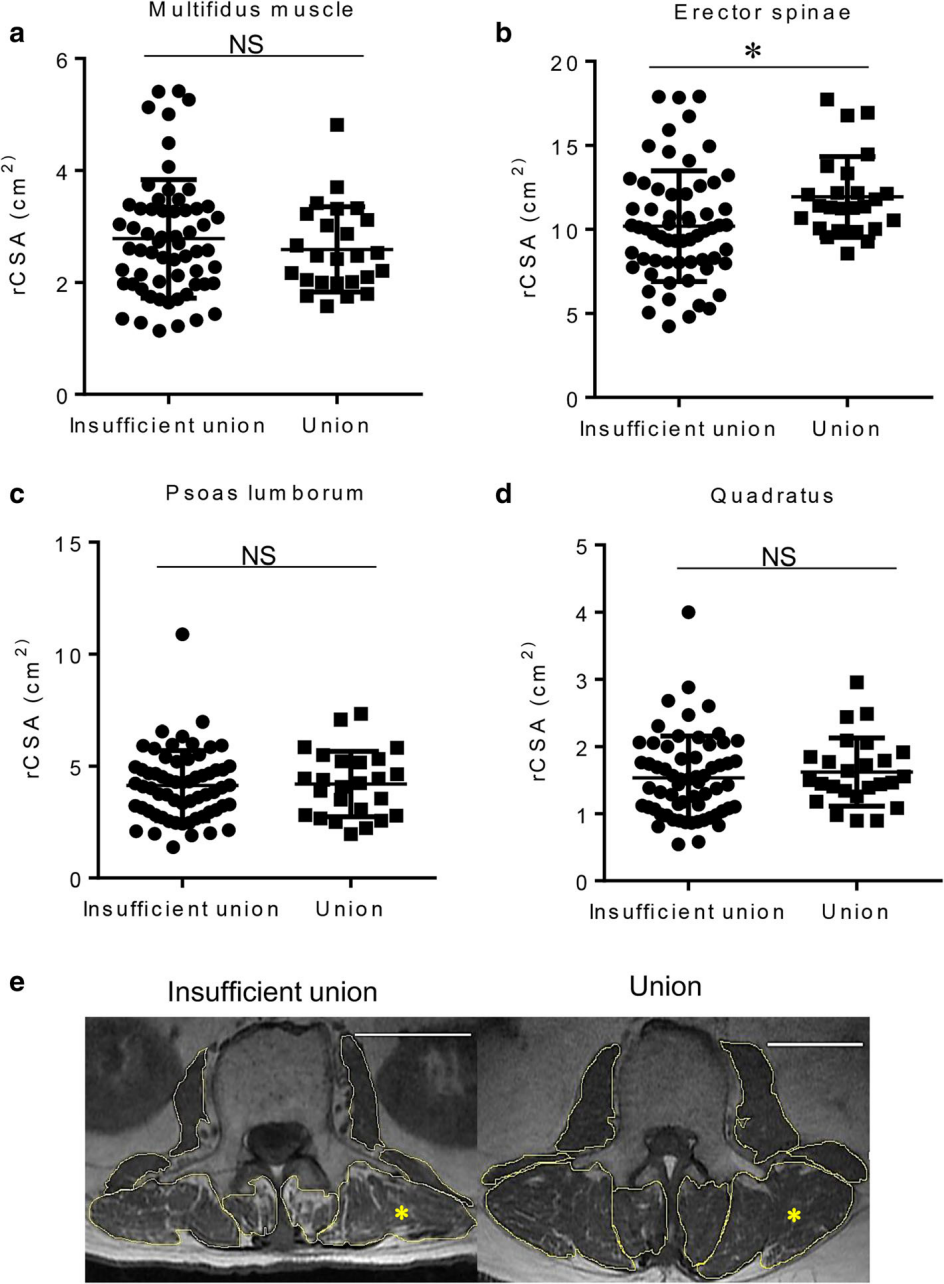
Graph comparing rCSA of the multifidus (**a**), erector spinae (**b**), psoas lumborum (**c**), and quadratus (**d**) between the groups (*P < 0.05, **P < 0.005, ****P < 0.0001, NS denotes no significant difference). **e** Representative images of paraspinal muscles of patients in both groups

To clarify the impact of paraspinal muscles on the outcome of conservative treatments of patients with OVF, we compared rCSA between the groups. rCSA of the erector spinae of patients in the group with sufficient union was significantly larger than the rCSA in patients in the group with insufficient union (Fig. 5b). No significant difference between the groups was found in the rCSA of multifidus, psoas, or quadratus muscles (Fig. 5a, c, and d). These results indicate that the rCSA of the erector spinae in patients with OVF who were treated successfully with conservative treatment is larger than in patients with OVF who had insufficient union (the paraspinal muscles of representative cases of both groups are shown in Fig. 5e).

## Discussion

The present study showed that rCSAs of total paraspinal muscles were positively correlated with BMD (YAM%) of patients with OVF (Fig. 2c). The timing of MRI after injury was not controlled because of the retrospective nature of the present study, but the average timing of MRI was 64.1 ± 13.71 days after the initial injury. We found a correlation between rCSA and FI% of paraspinal muscles and the timing of MRI after injury, and found FI% of the multifidus and erector muscles was positively correlated with the timing of MRI after initial injury. Interestingly, the rCSA of paraspinal muscles was not correlated with the timing of MRI after the initial injury. Based on this finding, we compared rCSA, but not FI, of paraspinal muscles in patients with insufficient union to the rCSA of patients with successful union. We found that the rCSA of the erector spinae of patients in the group with successful union was significantly larger than that in patients in the group with insufficient union.

MRI plays crucial role in evaluation of spinal pathologies [9]. Numerous studies have found an association linking morphological changes in paraspinal muscles with low back pain (LBP), lumbar spinal stenosis and spinal pathology using MRI [10, 12, 21]. Advancing age is associated with profound changes in the spine and its surrounding musculature, such as atrophied lumbar paraspinal muscles, with a reduction in their CSA and increased intramuscular FI [22]. Despite current study showed there was no significant difference LSS severity between groups, influence of morphometric change of paraspinal muscle by reduced innervation in LSS patients has been focused [12]. However, whether muscle control problems contribute to cause LBP, or whether LBP is a trigger for morphological changes in musculature, such as disuse muscle atrophy, remains largely unknown [21]. A recent report stated that profound changes in lumbar paraspinal muscles, a reduction in the CSA, and increased intramuscular FI occur in patients with osteoporotic spinal compression fractures [23]. However, this report lacked detailed information on the timing of the MRI assessments that were performed after injury. Therefore, we first examined the time-dependent changes of the paraspinal muscles of patients with OVFs to determine the influence of persistent back pain on paraspinal muscle morphology. We found a time-dependent increase in FI% of the multifidus and spinal erectors, but found no change in the rCSA of the paraspinal muscles of patients with OVF. These findings might suggest the mechanism underlying changes in muscle morphology in which fatty degeneration of the paraspinal muscles has occurred ahead of muscle atrophy (Fig. 4e). Further study using muscle biopsy during surgery is needed to elucidate the mechanism, which is likely related to the relationship between fatty degeneration and pathological muscle atrophy.

Despite the numerous studies in which efforts have been made to screen patients with an OVF who are at high risk of a poor prognosis [8, 24, 20], images of fresh vertebral fractures remain difficult to interpret. Recent studies have indicated the roles paraspinal muscles can play in OVFs, fracture prevention, interventions for management, rehabilitation after fracture, and modification of kyphotic changes after fracture [16, 25, 26]. We believe the present study has clinical importance because it shows that rCSAs correlate significantly with bone union; that is, larger rCSAs of spinal erectors can play a crucial role in reducing the risk of an insufficient union in patients with an OVF by contributing to spinal column stability in a protective way (Fig. 5e). Future mechanical study is needed to elucidate the reason for the significant contribution of the spinal erectors among all paraspinal muscles as found in the present study.

The present study has some limitations. First, because of the comparatively small sample size, data from men and women were analyzed together. Differences in the quality and size of paraspinal muscles by sex should be considered in future studies. Second, the retrospective study design was not conducive to obtaining control patients (no fracture) and MRI data from patients according to a structured routine. Third, measuring BMD at the femoral neck using DXA may not be representative of the mineralization of the axial skeleton, because osteoporosis in the peripheral and axial skeleton often develops disparately; thus, quantitative computed tomography of the lumbar spine should be used for further study. Last, conservative treatment was not fully standardized according to drug treatment including calcium/vitaminD, physiotherapy, brace type or the time the brace was worn as these items were selected by 3 physicians independently. The mechanism of fatty degeneration in the paraspinal muscles is presumed to be influenced by brace immobilization, the influence of persistent back pain, or degeneration as a result of muscle disuse, so these possibilities limit identification of the specific mechanism. In current study, 63% of the patients in insufficient group had severe fracture in Genant’s classification as compared to 20% in successful union group. This might be one factors the difference of fatty degeneration between groups. Further prospective studies, in which the timing of MRI after injury is controlled and conservative treatment is standardized, are needed to clarify the mechanism of fatty degeneration in the paraspinal muscles. Nevertheless, to our knowledge, this is the first report to determine that (1) fatty degeneration of the paraspinal muscles has occurred ahead of muscle atrophy in patients after OVF injury, and (2) larger rCSAs of spinal erectors can play a crucial role in in successful union in patients with OVF.

## Conclusions

These findings indicated a time-dependent increase of fatty degeneration of the multifidus and erector muscles, but no change in the rCSA and larger rCSAs of spinal erectors may play a role in successful union in patients with OVF.

## Additional file

Additional file 1: The intraobserver error of FI% of all patients. The reader evaluated and re-evaluated MRI, 2 weeks apart, blinded to MRI identifiers, to assess the intraobserver reliability of the rCSA and FI rate of paraspinal muscle measurement. (TIF 947 kb)
