# Supplementary figures and images for: Comparative study of the paraspinal muscles after OVF between the insufficient union and sufficient union using MRI
Source: BMC Musculoskelet Disord. 2018 May 14;19:143. doi: 10.1186/s12891-018-2064-0 (PMC5952377; doi:10.1186/s12891-018-2064-0)

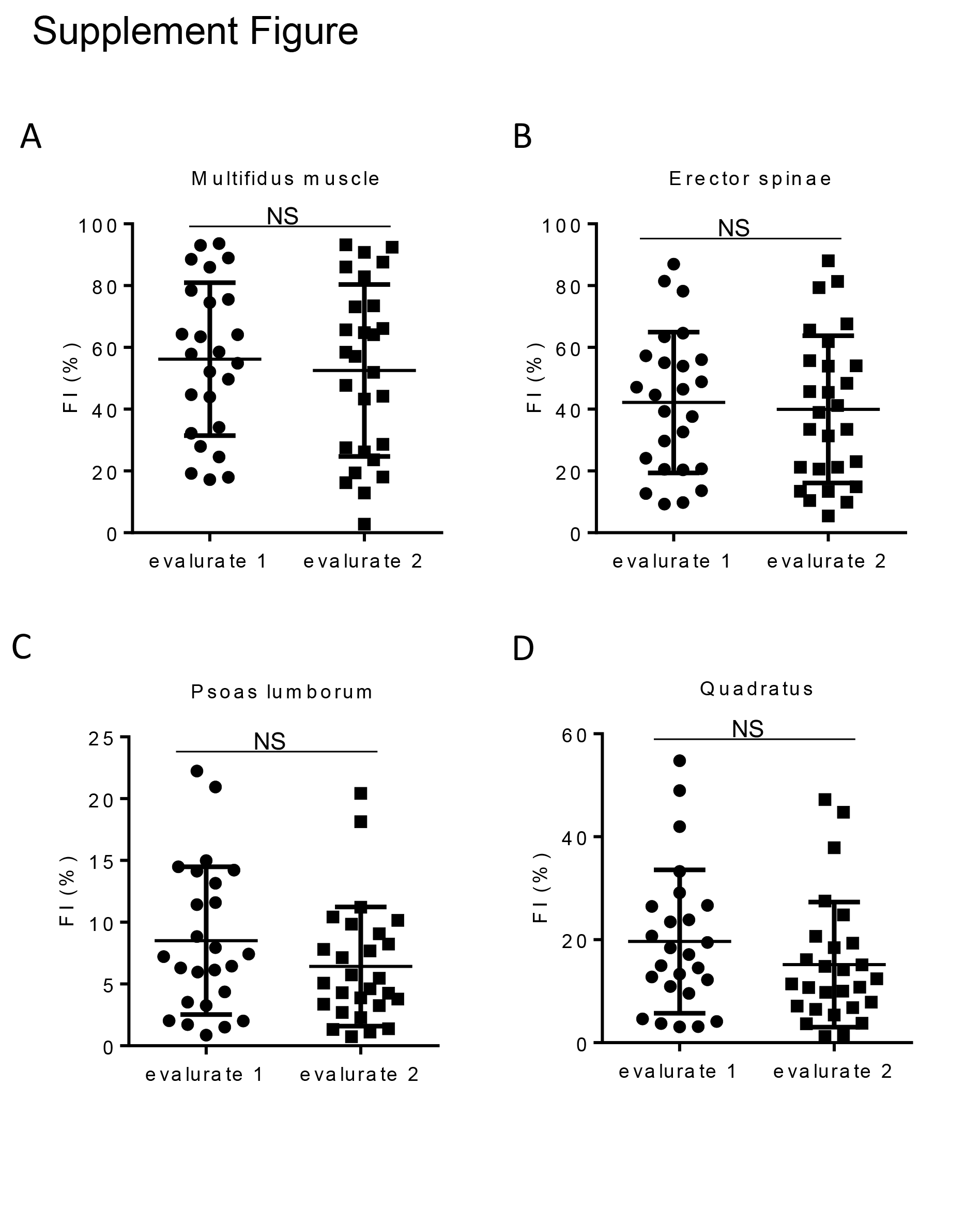

Supplement: Supplementary file 1 — The intraobserver error of FI% of all patients. The reader evaluated and re-evaluated MRI, 2 weeks apart, blinded to MRI identifiers, to assess the intraobserver reliability of the rCSA and FI rate of paraspinal muscle measurement. (TIF 947 kb) [file 12891_2018_2064_MOESM1_ESM.tif]
